# MENTAL HEALTH IMPACT OF BULLYING BY ETHNIC PEERS IN SENIOR HOUSING: A STUDY WITH OLDER KOREAN AMERICAN RESIDENTS

**DOI:** 10.1093/geroni/igae098.0136

**Published:** 2024-12-31

**Authors:** Yuri Jang, Juyoung Park, Min-Kyoung Rhee, Hi-Woo Lee, Nan Sook Park, Soondool Chung

**Affiliations:** University of Southern California, Los Angeles, California, United States; USC Suzanne Dworak-Peck School of Social Work, Los Angeles, California, United States; University of Southern California, Los Angeles, California, United States; Little Tokyo Service Center, Los Angeles, California, United States; University of South Florida, Tampa, Florida, United States; Ewha Womans University, Seoul, Republic of Korea

## Abstract

Bullying, a persistent negative interpersonal behavior directed at a specific individual or a group of individuals, is often associated with youth in school settings and young adults in the workplace. However, peer bullying is also common among older populations. Particularly in senior housing, where individuals with diverse backgrounds and characteristics reside in close proximity and where relocation is often not feasible, peer-to-peer aggressive behaviors are of concern. The purpose of the present study is to examine the experience of peer bullying and its mental health effects among ethnic minority older residents in subsidized senior housing. Surveys were administered to Korean Americans aged 65 and older (N = 343) in six senior housing facilities in the greater Los Angeles area. Multivariate regression models were used to examine the effect of being either a target or a witness of bullying on mental health outcomes, after controlling for covariates. About 18% of the sample had been a target of bullying, and over 31% had witnessed someone being bullied. Being a target of bullying was a significant predictor for both depressive symptoms and anxiety, whereas witnessing other residents being bullied was a significant predictor for anxiety only. These findings shed light on the common experience of bullying among ethnic minority older residents in senior housing as well as the adverse mental health impacts of peer bullying. Findings highlight the need for effective programs (e.g., awareness program, bystander education, and assertive communication training) to promote healthy interpersonal relationships in senor housing.

